# Androgenic-Induced Transposable Elements Dependent Sequence Variation in Barley

**DOI:** 10.3390/ijms22136783

**Published:** 2021-06-24

**Authors:** Renata Orłowska, Katarzyna A. Pachota, Wioletta M. Dynkowska, Agnieszka Niedziela, Piotr T. Bednarek

**Affiliations:** Department of Plant Physiology and Biochemistry, Plant Breeding and Acclimatization Institute—National Research Institute, Radzików, 05-870 Błonie, Poland; katarzyna.anna.pachota@gmail.com (K.A.P.); w.dynkowska@ihar.edu.pl (W.M.D.); a.niedziela@ihar.edu.pl (A.N.); p.bednarek@ihar.edu.pl (P.T.B.)

**Keywords:** androgenesis, barley, DNA methylation, MSTD, transposable elements

## Abstract

A plant genome usually encompasses different families of transposable elements (TEs) that may constitute up to 85% of nuclear DNA. Under stressful conditions, some of them may activate, leading to sequence variation. In vitro plant regeneration may induce either phenotypic or genetic and epigenetic changes. While DNA methylation alternations might be related, i.e., to the Yang cycle problems, DNA pattern changes, especially DNA demethylation, may activate TEs that could result in point mutations in DNA sequence changes. Thus, TEs have the highest input into sequence variation (SV). A set of barley regenerants were derived via in vitro anther culture. High Performance Liquid Chromatography (RP-HPLC), used to study the global DNA methylation of donor plants and their regenerants, showed that the level of DNA methylation increased in regenerants by 1.45% compared to the donors. The Methyl-Sensitive Transposon Display (MSTD) based on methylation-sensitive Amplified Fragment Length Polymorphism (metAFLP) approach demonstrated that, depending on the selected elements belonging to the TEs family analyzed, varying levels of sequence variation were evaluated. DNA sequence contexts may have a different impact on SV generated by distinct mobile elements belonged to various TE families. Based on the presented study, some of the selected mobile elements contribute differently to TE-related SV. The surrounding context of the TEs DNA sequence is possibly important here, and the study explained some part of SV related to those contexts.

## 1. Introduction

Plant tissue cultures are a well-established model to study distinct genetic [1,2] and epigenetic [3] changes related to abiotic factors that may be exhibited at the morphological level [4]. While DNA methylation pattern changes are linked to the Yang cycle’s proper functioning [5,6,7] or passive/active DNA demethylation [8,9], during cell reprogramming, i.e., oxidative modification of 5mC [10], it may be prone to point mutations [6,7]. Furthermore, DNA sequence changes may originate from the activation of retrotransposons [11] due to DNA methylation marks elimination [12].

Transposable elements (TEs) are the most common repeat sequences in the plant genome. TEs occupy from 3 to 85% of genomes [13,14] and, over millions of years, have increased plants’ genomes (such as maize or rice) [15,16]. Systematics of TEs distinguish classes, subclasses, orders, superfamilies, families, and subfamilies [17]. Transposable elements are divided into Class I, which includes retroelements (retroviruses and retrotransposons) and class II, encompassing DNA transposons. Phylogenetic analyses based on reverse transcriptase amino acid sequences resolve the Long Terminal Repat (LTR) retrotransposons into families: the Ty3-*gypsy* retrotransposons (*Metaviridae*) and the Ty1-*copia* elements (*Pseudoviridae*) [18,19,20]. The Ty1-*copia* and Ty3-*gypsy* retrotransposons represents 70% and 20% of all *Triticeae* TEs superfamilies, respectively [21]. Another group of LTR retrotransposons described, termed terminal-repeat retrotransposons in miniature (TRIM), are present in plants [22]. These elements cannot transpose autonomously, and require the assistance of mobility-related proteins encoded by other retrotransposons [23]. TRIMS have been identified in the genomes of cereals such as rice [24] and barley [23,25]. In addition to the TRIM group, there is the large retrotransposon derivatives (LARD) group of non-autonomous retrotransposons also identified in barley genomes [26]. Transposons, similar to retrotransposons, have been divided into several families. One is called the CACTA, which received its name as it is flanked by inverted repeats that terminate in a conserved CACTA motif. The CACTA family was identified inter alia in soybean [27], maize [28], or barley [29,30].

The cell differentiation due to hormonal stimulation [31], favors the formation of genetic [32] and epigenetic [33] changes under in vitro conditions. De novo methylation and DNA demethylation processes initiate silencing or the activation of TEs in the callus [34,35] or the regenerated plants [11]. Not all TEs are activated under in vitro tissue culture environment [36,37,38]. There are many reasons for this. For example, some TEs are highly methylated [39] and, consequently, are not active. DNA methylation of such regions is due to epigenetic mechanisms recognizing regions rich in repeated sequences including TEs [40,41]. The others, possibly those that persisted in the genome for a long time, accumulated point mutations and became inactive [42]. Alternatively, the activity of TEs missing the sequence responsible for transposition might be limited [43]. Furthermore, TEs affecting genome functioning are under selection pressure [44]. Transposable elements behave as effective mutagens that lead to a genetic variation at the insertion loci. An arising mutation can be neutral, lethal, or valuable for the host organism. Those which are lethal are removed during evolutionary pressure; the neutral and beneficial may settle in genomes [45]. Therefore, one may expect that TE families negatively affecting genome functioning should be inactivated or even eliminated in plants [46]. Evidently, however, retro- or transposon migration is one of the many reasons underlying the in vitro induced variation observed in regenerants [47]. Under in vitro plant regeneration, the activation of retrotransposons and DNA transposons was demonstrated. Among them are *ONSEN* (Ty1-*copia*—like retrotransposon) [11] and LORE1 (*Lotus* retrotransposon 1, belonging to the Ty3-*gypsy* group of elements) [48] and the transposon belonging to the *h*AT superfamily (class II DNA transposons) in the promoter region of *flavonoid 3′, 5′-hydroxylase* (*F3′5′H*) which is related to anthocyanin synthesis [49].

Despite numerous reasons to expect the presence of in vitro induced TE-dependent variation, the relation of TEs’ activity and the input of distinct TEs families into in vitro tissue culture-induced sequence variation is not apparent. It is not obvious to what extent TEs activity is regulated epigenetically or whether DNA sequence methylation context is essential. The methylated cytosine (5-methylcytosine) is associated with numerous biological processes such as inactivation of transposable elements [50], imprinting genes involved in flowering [51], or adaptive response to environmental stresses [52,53]. DNA methylation pattern alternations may manifest in developmental abnormalities in plants, such as short plant stature [54], altered leaf size and shape, decreased fertility, altered flowering time [55,56], or resulting in abnormal seeds and seedling lethality [57]. In plants, two symmetrical CG, CHG, and one asymmetric CHH (where H can be A, T, or C) [58] contexts were evaluated. Different methylation contexts have various mechanisms to maintain methylation [59,60,61,62] or introduce it de novo [63]. However, demethylation can be passive [8] or active [9]. The relationship between the two opposed phenomena and the involvement of many cellular processes may distinctly influence the activity of TEs families.

Various methods have been used to study the genome changes caused by TEs activity. First of all, these were methods using PCR: IRAP (Inter-Retrotransposon Amplified Polymorphism (IRAP) [64,65], Retrotransposon-Microsatellite Amplified Polymorphism (REMAP) [64,66], and Sequence-Specific Amplified Polymorphism (SSAP) [67]. While IRAP, REMAP, or SSAP can estimate sequence changes, the Methyl-Sensitive Transposon Display (MSTD) technique [68] offers the opportunity to study changes caused by retrotransposon activity and alternations in DNA methylation. The MSTD method enables simultaneous analysis of changes related to TEs movement and DNA methylation pattern alternations. Its application is still limited by the restriction enzymes (*Msp*I and *Hpa*II) which do not identify all sequence contexts [69]. The MSTD variant, based on the metAFLP technique dedicated to studying plant materials from tissue cultures [70,71], does not have such limitations, except for the opportunity to study global DNA methylation level. The latter could be analyzed using RP-HPLC approach [70,72,73,74].

The study aims to evaluate the role of particular mobile elements belonging to selected TE families and DNA methylation/demethylation of sequence contexts and donor plants impact on sequence variation (SV) originating under anther tissue culture of barley.

## 2. Results

### 2.1. Visual Inspection of Plant Material

The regeneration of barley plants via androgenesis in anther culture resulted in both green and albino plants. As albinos were not subjected to study, the final number of green regenerants (R) which were fertile doubled haploids (DH) from four donor plants (D68, D69, D70, and D72) amounted to 80 regenerants (20 plants for each donor plant). DH regenerants from successive donor plants (D68–D72) were grouped and named R68, R69, R70, and R72, respectively. All regenerants were similar in morphological traits (leaf height, width, or method of tillering) with donor plants and showed no differences among themselves. DNA isolation resulted in samples of expected purity and integrity.

**Figure 1:**
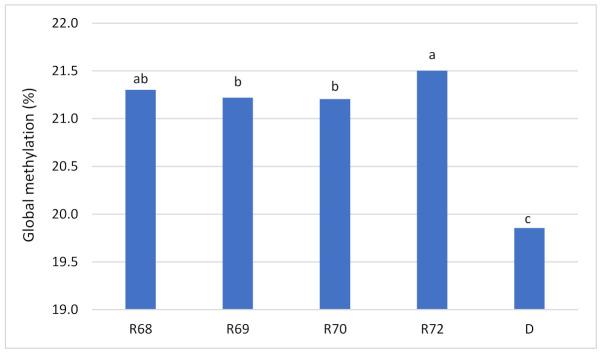
DNA methylation content (global methylation) based on RP-HPLC analyses. The a, b, c, Games–Howell post hoc test grouping. D-donor plants grouped together; R68–R72-regenerants obtained from successive donor plants.

The RP-HPLC results demonstrated that the global genomic DNA methylation score increased from donor plants (19.85%) to regenerants (21.21–21.50%). A one-way ANOVA conducted on the RP-HPLC-based level of global DNA methylation showed that donors and regenerants differed at the DNA methylation level [*F*(4,20.918) = 62,840, *p* < 0.0001]. The Games–Howell post hoc analysis revealed that donors formed a distinct homogenous subset (c). The R68, R69, and R70 formed the second (b), whereas the R68 and R72 formed the third subset (a), with donors being in the subset (c) (Figure 1).

### 2.2. MSTD Profiling

**Figure 2:**
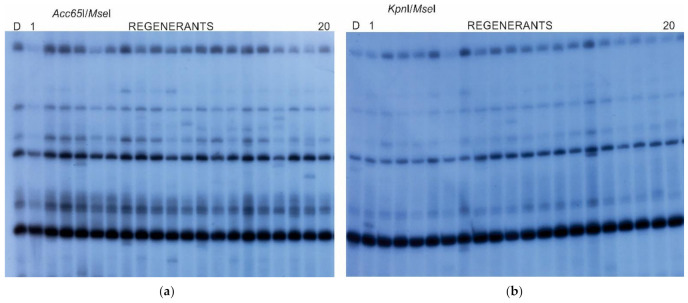
An example of Methyl-Sensitive Transposon Display (MSTD) profile generated with *BAGY-1*-C2043 and CpG-GCA selective primers. The *Acc65*I/*Ms*eI (**a**) and *Kpn*I/*Mse*I (**b**) reflected DNA profiles obtained with various restriction enzymes. Line D represents DNA profiles of the donor, lines 1–20 DNA profiles of the same regenerants obtained from different digestions.

MSTD profiling of regenerants and donor plants (Figure 2, File S1) with 17 selective primer combinations identified 706 markers shared between the *Kpn*I/*Ms*eI (K) and *Acc65*I/*Mse*I-*Kpn*I/*Mse*I (M) platforms. The number of polymorphic bands for the K and M markers amounted to 458 and 521, respectively.

The percentages of polymorphic loci (*%P*) for donors in K and M platforms were 30.88% and 47.03%, respectively (Table 1). The parameters related to *%*P for regenerants obtained from the various donor plants ranged from 38.81 to 41.93 for the K platform and from 52.97 to 56.94 for the M data set. When Shannon’s information index (*I*) was taken into account for donor plants, its values amounted to 0.167 for the K and 0.248 for the M markers. Shannon’s information index (*I*) for regenerants ranged from 0.128 to 0.141 for the K and from 0.220 to 0.230 for the M data (Table 1).

### 2.3. Molecular Characteristics Based on MSTD Data

The total tissue culture-induced variation (TCIV) based on MSTD data related to all regenerants used in the analysis was 23.15%. The following molecular characteristics that create total TCIV were multifaceted, and ranged from 1.68% for de novo methylation (DNMV), through 3.62% for demethylation (DMV), to 12.34% for sequence variation (SV). Considering TCIV, the extreme values belonged to R69, R72, and R70 (Table 2). Analysis of in vitro induced variation for different elements belonging to various families of transposable elements showed significant differences between them. The highest value of TCIV was observed for *Cassandra* (TRIM) (33.32%), and the lowest for *Sukkula* (LARD) and *BARE-1* (Ty1-*copia*) (about 14%) (Table 2).

### 2.4. Analysis of Variance

Two-way ANOVA based on sequence variation (SV) evaluated that using the metAFLP approach, assuming the presence of interaction of regenerants derived from four donor plants and various mobile elements from different TE families, as well as main effects, was significant (*F*(19,380) = 85.114, *p* < 0.0005, R^2^*_adj_* = 0.8). An interaction between four regenerant groups and different mobile elements from various TE families was significant (*F*(12,380) = 4.017, *p* < 0.001), and explained 11.3% of the variance in the sequence variation (*η*^2^ = 0.113).

Simple main effects analysis showed differences in sequence variation means for the regenerant groups in the case of all analyzed mobile elements (Table 3). When the Bonferroni correction was applied (α = 0.05/20 (twenty simple effects: four groups of regenerants derived from different donor plants multiplied by five mobile elements belonging to five TE families) = 0.0025), then regenerants differed in SV for *Cassandra* (TRIM), *BARE-1* (Ty1-*copia*), and *BAGY-1* (Ty3-*gypsy*). The explained variance varied from 7.6 to 16.3%, as indicated by partial *η*^2^ values (Table 3).

**Figure 3:**
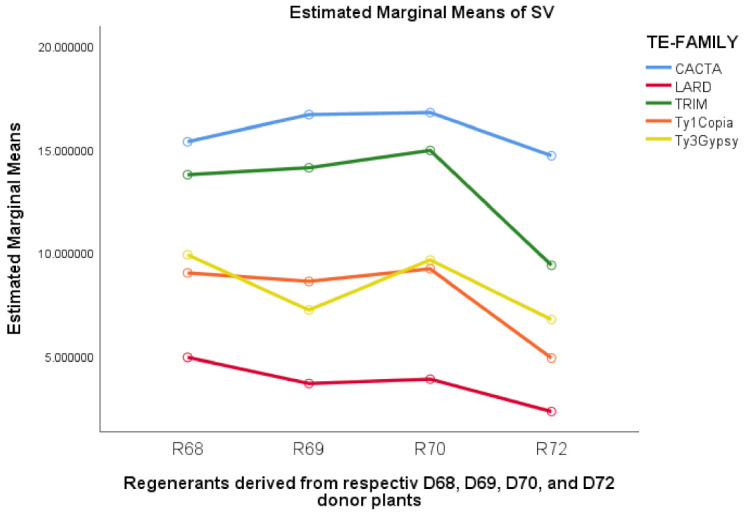
Estimated marginal means of sequence variation (SV) for mobile elements belonged to five TE families within each of the regenerant groups derived from respective donor plants.

Analysis of simple main effects concerning sequence variation, by means of mobile elements from various TE families for the regenerant groups derived from distinct donor plants, demonstrated that such differences were all significant (Table 4) when Bonferroni correction (0.0025) was applied. The explained variance varied from 41.6 to 53.8%, as indicated by partial *η*^2^ values (Table 4). The highest SV values were generated by *Balduin* (CATCA), whereas the lowest by the *Sukkula* (LARD) mobile elements, independent of the regenerant group analyzed (Figure 3). In general, SV levels for all regenerant groups and the given mobile elements were similar, with the lowest values in the R72 group.

**Figure 4:**
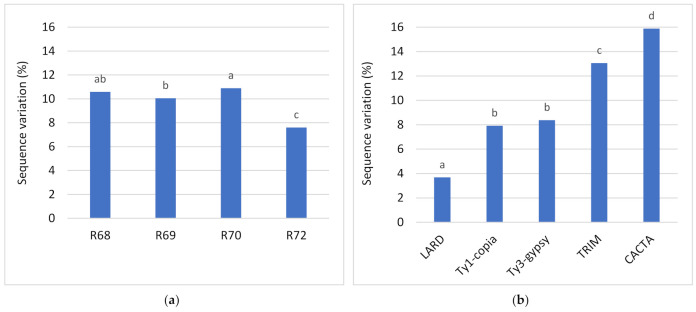
Homogeneous subsets of (**a**) regenerant groups derived from donor plants and (**b**) mobile elements belonging to five TE families based on SV and evaluated using the Ryan–Einot–Gabriel–Welsch Range post hoc tests. R68–R72-regenerants obtained from successive donor plants. *Sukkula* (LARD), *BARE-1* (Ty1-*copia*), *BAGY-1* (Ty3-*gypsy*), *Cassandra* (TRIM), and *Balduin* (CACTA)—mobile elements belonging to families of transposable elements.

Based on the Ryan–Einot–Gabriel–Welsch Range test (REGWR), the regenerants derived from the donor plant D68 and D69 (subset a), D68 and D70 (subset b), and D72 (subset c) composed three separate groups based on the mean values of SV (Figure 4a). Similar analyses for mobile elements belonging to various TE families showed that *BARE-1* (Ty1-*copia*) and *BAGY-1* (Ty3-*gypsy*) mobile elements were in the same homogeneous subset (subset a), whereas the other elements formed three other separate groups (subset b, c, and d) (Figure 4b).

### 2.5. Linear Regression

Linear regression model testing as to whether global DNA methylation derived based on RP-HPLC analysis explains that the SV, evaluated based on MSTD, was non-significant (*F*(1,398) = 0.255, *p* = 0.614).

Multiple regression was run to predict CHH_SV from CHH_DMV, CHH_DNMV, and CHH_DMV*CHH_DNMV (A), CHG_SV from CHG_DMV, CHG_DNMV, and CHG_DMV*CHG_DNMV (B), as well as CG_SV from CG_DMV, CG_DNMV, and CG_DMV*CG_DNMV (C). The independence of residuals, as assessed by Durbin–Watson statistics, was assumed for B (d = 1.53) and C (d = 1.62), but not for A (d = 0.69). Therefore, coefficients of A should be treated with caution. Other assumptions of multiple regression (linearity, homoscedasticity, multicollinearity, and normality) for B and C were not violated. The regression models A (*F*(3,396) = 27.514, *p* < 0.0005, R^2^*_adj_* = 0.166), B (F(3,396) = 25.614, *p* < 0.0005, R^2^*_adj_* = 0.162), and C (*F*(3,396) = 32.551, *p* < 0.0005, R^2^*_adj_* = 0.198) were significant. Interestingly, in all contexts B coefficients of DNMV were higher than those for DMV. The lowest B values were detected for interactions. When standardized coefficients were analyzed, values for DMV were higher than for DNMV, and interactions were the lowest. It should be stressed that all models exhibited comparable percentages of variance with slightly higher values for CG contexts. The regression coefficients and standard errors can be found in Table 5.

Regression analysis was performed to verify whether DMV and DNMV of distinct sequence contexts predict SV within those contexts for each used mobile element, separately. Eight models were significant; however, only in *Sukkula* (LARD) and *BAGY-1* (Ty3-*gypsy*) mobile elements were some regression coefficients significant. The CHG_SV for the *Sukkula* (LARD) element was predicted by CHG_DNMV. In the case of *BAGY-1* (Ty3-*gypsy*), SV was predicted by CHH_DMV and CHG_DNMV. It should be stressed that, in the case of the *Sukkula* element, Durbin–Watson statistics were below 1.5, and the assumption of regression was violated (Table 6).

## 3. Discussion

Plant regeneration via anther cultures is subjected to somaclonal variation [75], or tissue culture-induced variation [76], manifested at the level of plant morphology, genotype, or both simultaneously. Often, these different terms describe the same phenomenon and can be used interchangeably [77]. Such variation is due to stressful conditions that accompany regeneration beyond normal plant development and growth. In the presented experiment, the plants derived via anther culture were identical in shape to donor plants. Nevertheless, the lack of phenotypic changes does not prove that the regenerated plants are identical in DNA methylation pattern and DNA sequence. The RP-HPLC data demonstrated that DNA methylation increased in R compared to D plants. The result is fully congruent with barley data [78] and *Gentiana pannonica* Scop. [70]. Interestingly, the presented direction of DNA methylation level change is not always the same. In some instances (i.e., triticale) a decrease in DNA methylation was demonstrated [79]. It is not evident why, in some cases, the methylation level increases whereas decreases in others. A suggestion could be ploidy level; the notion could be supported as barley and *Gentiana pannonica* Scop have 2n genome, whereas triticale is hexaploidy [80]. Another alternative is genome stability. At least in triticale, a synthetic species with a relatively unstable genome, various changes are quite common [81], and DNA methylation may be a key factor responsible for such instability. It also cannot be excluded that changes in global DNA methylation are associated with nuclear DNA changes. The increase in the total DNA amount was detected among others in *Nicotiana sylvestris* selfed DH progenies [82]. However, in the presented work, we analyzed the DNA of regenerants (DH), i.e., plants obtained directly from in vitro culture without undergoing the generative cycle. On the other hand, an increase in genomic DNA methylation may be related to repeat sequences’ methylation [83]. However, RP-HPLC does not allow the verification of this hypothesis.

The MSTD approach proved to be informative for *Kpn*I/*Mse*I and *Acc65*I/*Mse*I—*Kpn*I/*Mse*I platforms, as indicated by Shannon’s *I* indexes. It should be stressed that the *Acc65*I/*Mse*I—*Kpn*I/*Mse*I data reflecting DNA methylation variation were more informative than the *Kpn*I/*Mse*I detecting sequence variation only. Hence, the two marker types evaluated based on the MSTD platform could be applied for estimating the quantity of tissue culture-induced variation.

It is suggested that explant tissue donor plants might impact sequence variation exhibited among regenerants [2,6]. Similarly, sequence variation might depend on point mutations [84], but also on the transposition of retrotransposons [11] that may take place during cell reprogramming [85]. In addition, few studies show the level of sequence variation associated with mobile elements belonging to different classes of TEs concerning plant regeneration by in vitro cultures. What is known is the level of polymorphism identified, for example, by the IRAP technique based on primers designed for the *BAGY-1* [86,87,88] or *BAGY-2* [89] mobile elements in barley callus, or in *Dendrobium nobile* [90]. It is also possible that interaction between a donor and selected mobile elements belonging to various TE families may be crucial for sequence variation. The two-way ANOVA demonstrated the interaction between regenerant groups derived from distinct explant source donor plant and the mobile elements belonging to five TE families. Although significant, the percentage of variance explained by such an interaction was relatively low, reaching 11.3% of SV. Analysis of simple main effects demonstrated that particular mobile elements from various TE families differed in SV’s mean scores, depending on regenerant groups obtained from various donor plants. Interestingly, independently of some variation in SV for the given mobile element, as indicated by the estimated means of sequence variation, SV’s general behavior was similar to that shown in Figure 2. Primers based on *Balduin* element (CACTA) and on *Cassandra* (TRIM) [22] generated the highest, whereas based on *Sukkula* element (LARD) [26] and on *BARE-1* (Ty1-*copia*) [53], the lowest values of SV were generated. CACTA transposable elements belonging to DNA transposons are ubiquitous in plants [91]. This TE family may capture cellular genes, replicate and transport them to other regions of the genome [92], and create a new functional gene by rearranging gene fragments [93]. Additionally, the presence of CACTA in AT-rich regions suggests a high tendency for insertions and generating changes [94]. Hence, this may be why mobile elements belonging to these families exhibited the highest sequence variation in the presented work. On the other hand, a slightly lower level of SV created by *Cassandra* (TRIM) compare to *Balduin* (CACTA) may be explained by TRIMs lower amplification rate than MITEs (e.g., CACTA), reflecting the diverse transposition mechanisms of retrotransposons and DNA transposons [95].

An interesting issue of the study is the activity of different TE families. The activity may rest on, i.e., their degeneration level and, thus, the ability to switch from one position in the genome to the other. Assuming that such degeneration is related to the rate of mutations, which varies between species and issues [96], then the TEs mobility might be related to the moment they inhabited the species [97,98]. Unfortunately, such data is hardly available, making this hypothesis difficult to verify. An alternative option may rely on the TEs surrounding sequence, or on the level of methylation/demethylation that proceeds during plant regeneration. It was suggested that, during plant regeneration, genomic DNA needs to undergo demethylation [62,99] followed by de novo methylation [100]. It is stated that pollen reprogramming to embryogenesis is associated with the decrease in global DNA methylation which is necessary for the acquisition of embryogenic competence by the microspores [101]. The regeneration process may proceed differently in distinct species and may depend on whether anther or zygotic embryo cultures are applied. In triticale plant regeneration via anther culture, DNA demethylation is not re-established, even after several generative cycles [79]. In barley, however, the DNA in regenerants has a higher level of methylation than the donor plants, and the level of such methylation remains constant after a single generative cycle [78].

Independently of the donor plant used as a source of explants, the regenerants groups differed in terms of SV. The mean SV for D70 and D68 derived regenerants were close to one another. The same was observed for D68 and D72 derived regenerants (Table 2). ANOVA showed that regenerant groups explained up to 53.8% of SV variance related to TEs, suggesting that even highly related genotypes may have different input in SV generated by mobile elements belonging to various TEs.

It was suggested that TEs mobility (and consequently SV) is related to DNA demethylation level [102,103,104] and, thus, to the cell reprogramming stage [105]. Studies on global DNA methylation of genomic DNA evaluated based on RP-HPLC analysis revealed that mean scores of genomic methylation of regenerant groups were higher than the donor plant group, and that R72 differed from all the other groups of regenerants. In contrast, DNA methylation’s respective values for R70, R69, and R68 were at a comparable level. However, regression analysis failed to link DNA methylation changes evaluated based on RP-HPLC and SV characteristics. Such a result is the consequence of the RP-HPLC analysis itself. The approach can identify robust effects, but not subtle ones which may be vital here. It should be taken into account that the data obtained from the RP-HPLC do not show changes in demethylation or de novo methylation, but only the result of these two opposite processes. Moreover, not every methylation change is associated with sequence variation. More detailed information on SV is provided by multiple regression.

To test whether DNA demethylation, or de novo methylation affecting varying DNA sequence contexts, may explain TE-related SV within respective contexts, the MSTD was used. The regression analysis demonstrated that, to some extent, SV could be explained by DNA demethylation and de novo methylation (Table 6). It was established that all symmetric and asymmetric contexts were essential, but they explained a small SV fraction. This may suggest that reasons other than DNA methylation contribute to TE-dependent SV. The low input of DNA methylation characteristics may suggest that the presented experiment either failed to capture a considerable amount of methylation changes that appeared during cell reprogramming, or that such changes were sufficient to activate some mobile elements belonging to analyzed TE families. At the same time, the other mobile elements activity might not have depended on DNA methylation.

The presented analysis is based on regenerants derived via anther culture. Therefore, it is not possible to analyze phenomena during the earlier stages of plant regeneration. The analyzed individuals survived regeneration and probably have an acceptable level of changes allowing them to function. Such a hypothesis is in line with that proposed earlier, where it was suggested that only plants with an acceptable level of changes might regenerate and survive [106,107]. However, it cannot be excluded that a small input of methylation changes into SV reflects a real phenomenon. It is well documented that epigenetic processes are very subtle, and even tiny DNA sequence context methylation changes might be sufficient for the activation of some TEs [108].

Regression analysis concerning the role of DNA methylation changes explaining SV related to specific DNA sequence context performed for each mobile element from various TE families independently demonstrated that the mobile elements with the highest values of sequence variation (*Balduin* and *Cassandra*) were not associated with any of the DMV and DNMV characteristics associated with respective sequence contexts. The TEs were identified mainly in AT reach regions [109], which supports presented findings that methylation is not a critical factor in controlling their migration. It is worth noting that, in barley regenerants, mobile element *Balduin* seems to not contribute to plant morphology, which is also the case in the presented experiment, and such an effect was also observed in other species [110]. The high level of SV that originated from the MSTD profiles based on *Cassandra* is not surprising, although it is confusing. The TRIM family lacks autonomous sequences that allow independent transposition. Still, they can transpose in trans [23]. The small TRIM size, and their less harmful effects during moving to genic region than large TEs, increase the probability that their insertions are preserved [95]. The lowest mean sequence variation (CHG_SV) was identified when the *Sukkula* sequence was used and was due to CHG_DNMV. The family’s activity is possibly well controlled by de novo methylation of CHG contexts, leading to decreased sequence variation induced by that transposable element.

Similarly to the TRIM family, LARD originated from degenerated LTR elements [111]. They are non-coding structures with intact termini with no opportunity to move without the assistance of other autonomous TEs [112]. Possibly, that lack of opportunity to move and the fact that LARD TEs seem to be under CHG_DNMV control resulted in the lowest values of SV among all analyzed TE families.

Ty1-*copia* elements are probably the most abundant among LTR retrotransposons. Their sequence variations can be used as a molecular clock of insertion [113]. Ty1-*copia* elements are more often linked to genes than Ty3-*gypsy* elements [114]. Ty1-*copia* may alter gene regulation [115], induce transduction events [116], or lead to epigenetic silencing [117]. Hence, *BARE-1* migration may result in SV affecting plant functioning. Under tissue culture conditions, their mobility (reflected at the SV) was lower than that for *BAGY-1*. These results demonstrated that *BARE-1* might not be under methylation control, whereas CHH_DMV, CHG_DMV, and CHG_DNMV control *BAGY-1*. The reason the *BAGY-1* element generates higher SV than *BARE-1* element (Ty1-*copia*) is not apparent. Most probably, this may reflect the ability of Ty1-*copia* to affect gene functioning and genome structure.

Presented data demonstrate that CHH and CHG contexts are affected less than CG by SV related to chosen mobile elements activity. This contrasts with results for *Arabidopsis thaliana*, maize, and olive palm [118]. Such a discrepancy may be explained either by differences in the species analyzed or the used molecular approach. Maybe the restriction sites for *Kpn*I-*Acc65*I are distributed unevenly along chromosomes, or are distinctly represented in hetero and euchromatin, leading to biased results.

It is worth mentioning that, despite a high level of SV related to analyzed mobile elements from various TE families and donor plant effects were revealed in the study, no evident morphological changes of regenerants were evaluated in the presented study. This may suggest that, at least in the barley genome, either the chosen mobile elements’ movement do not affect vital cell functioning, or that significantly affected microspores cannot switch their fate and/or cannot regenerate plants. The CHH_DMV, CHG_DMV, and CHG_DNMV characteristics were important in explaining respective TE-dependent SV (*BAGY-1* and *Sukkula*). Assuming the CHH and, to some extent, the CHG methylation contexts may be under epigenetic control, it could be thought that epigenetic processes induced by in vitro plant regeneration are crucial here. However, it is surprising that the contexts could have explained only a minor part of TEs and donor-dependent variation. Possibly, analyzing SV changes at the regenerant level hides phenomena that take place at earlier stages of plant regeneration and those stages depend on DNA methylation.

The presented study has obvious limitations. Using the MSTD approach, the evaluated markers may not necessarily reflect changes affecting transposon and retrotransposon sequences, as one of the selective primers (the one complementary to the *Acc65*I/*Kpn*I restriction site) may not be present within the sequence. We cannot exclude that methylation changes reflect genomic regions surrounding mobile elements. Our results concerning methylation changes might be interpreted in terms of changes affecting surrounding sequences in this context. Then, it is not evident whether such results should be interpreted concerning methylation of the mobile elements or their mobility. On the other hand, the level of methylation (and sequence) changes evaluated in the study is only slightly higher than that for the same species using metAFLP alone [76,119,120]. Assuming MSTD amplifies short fragments (the range is 45–500 bp [121]), our results may, at least partly, reflect mobile elements’ movement due to methylation changes due to microspore reprogramming. The problem could be solved via sequencing some of the markers and verification whether amplified sequences reflect mobile elements and to what extent they reflect their nearest vicinity. However, direct sequencing of the MSTD and AFLP fragments is usually complicated, as a single band may be composed of multiple fragments [122]. In addition, to verify whether DNA methylation changes affect mobile elements, sequencing would require primers from the *Acc65*I/*Kpn*I site, which is not easily available. Unfortunately, such analyses were not possible within the study.

It could also be speculated that, using the MSTD approach, one cannot identify mobile element movement unless a study is conducted on a single cell. However, our plant materials were prepared in such a way that each regenerant was expected to regenerate from a single microspore. Thus, we tend to think that the current study design is adequate for studying tissue culture-induced mobile element mobility and TCIV that might be due to their activity.

Although transposable elements may induce SV, it seems this has little importance for large scale production of DH plants. Usually, regenerants that differ in type with the donor of explants can be easily removed from breeding programs. The presented study demonstrates that identified changes may have scientific implications allowing better understanding of genome functioning. Furthermore, knowledge on how in vitro culture conditions affect regenerants’ variation may be important when additional variation is needed and, for example, application of GMO is not prohibited.

## 4. Materials and Methods

### 4.1. Plant Material

Analyses were performed on one spring barley cultivar NAD2 (Poznań Plant Breeders LTD, Nagradowice, Poland). Leaves from four donor plants (D68, D69, D70, and D72) and eighty regenerants (R) were used to determine tissue culture-induced variation (TCIV) using transposable element markers. Donor plants (D) were offspring of barley regenerants obtained via anther cultures described previously [123]. Donor plants were grown in controlled conditions (photoperiod: 16h light/8 h dark, temperature: 16 °C/day and 12 °C night) until picking spikes. The microspore developmental stage was determined by squashed anthers in a drop of aceto-carmine solution (2%) and observation under microscope. Tillers with anthers containing the majority of microspores at the mid- and late-uninucleate stage were collected and surface-sterilized in 70% ethanol for 1 min and then in 10% sodium hypochlorite for 20 min. Anthers were excised under sterile conditions and located in 3M mannitol solution with 2.5 mg L^− 1^ CuSO_4_ × 5H_2_O [124] for 5 days in 4 °C as pretreatment conditions [125]. After pretreatment, anthers were transferred onto solid induction medium KBP with 0.9 mg L^−1^ 6-benzylaminopurine (BAP) [126] with modification [127]. Petri dishes with explants were incubated in the dark at 26 °C. After ca. 3 weeks, the first calli and embryos were transferred onto regeneration medium K4NB with 0.025 mg L^−1^ BAP [126] with modification [127]. Androgenic structures were kept under a light regime of 16 h day/8 h night at 26 °C. Green regenerants were transferred to flasks containing rooting medium N6I [128] supplemented with 2 mg L^−1^ indole-3-acetic acid (IAA). Subsequently, the developed seedlings were transferred to pots and grown in a greenhouse. The chromosome number doubled spontaneously. Plant morphology was estimated, taking into account the plant height and leaf shape.

In summary, four donor plants—D68, D69, D70, and D72—were used in the analysis. If all of them were analyzed together, they formed one group called D.

Eighty regenerants (R) were obtained. Regenerants from individual donor plants were grouped, and four groups of 20 individuals were created—R68, R69, R70, and R72.

### 4.2. Leaf genomic DNA Extraction

The genomic DNA was isolated by grinding 100 mg leaves from young regenerants frozen in liquid nitrogen using DNeasy MiniPrep kit (Qiagen). The concentration and purity of isolated DNA samples were evaluated using ND-1000 spectrophotometer (Thermo Fisher Scientific). The integrity of DNA was verified spectrophotometrically in 1.2% agarose gel.

### 4.3. Reverse Phase-High Performance Liquid Chromatography

DNA samples were prepared for reverse phase-high-performance liquid chromatography (RP-HPLC) analysis according to previous studies [78]. The amount of global DNA methylation was calculated as the concentration of 5-methyl-2′-deoxycytidine (5mdC) in relation to the whole amount of cytidine according to the formula: 5 mdC/(5 mdC + dC) × 100. Mean values and standard deviation of the amount of global DNA methylation were evaluated for donors and regenerants. All detailed results have been included in the Excel spreadsheet (File S1).

### 4.4. Methyl-Sensitive Transposon Display

The Methyl-Sensitive Transposon Display (MSTD) approach was based on the metAFLP procedure [120]. The DNA samples were divided and digested with two sets of endonucleases: *Acc65*I and *Mse*I; and *Kpn*I and *Mse*I. The digestion step was followed by adaptor ligation, pre-selective, and selective amplification steps. After the ligation step, the reaction mixtures were diluted with water (1:3, *v*/*v*). The pre-selective PCR product before selective amplification also was diluted (1:20, *v*/*v*). For the selective amplification step oligonucleotides directed towards mobile elements belonged to five families of transposable elements sequences and *Kpn*I/*Acc65*I adaptor sequences were used (Supplementary Table S1).

Selective amplification was conducted in the presence of ^32^P-labelled selective primers. After fractioning the selective PCR product with 7% polyacrylamide gel electrophoresis (PAGE), DNA fragments were visualised by exposing to X-ray film.

After DNA banding profiles were obtained, DNA markers assigned to sequence changes and DNA methylation changes were extracted based on the procedure described earlier [129]. DNA band profiles obtained for digestion with *Acc65*I and *Mse*I (A) and *Kpn*I and *Mse*I (K) were collated and scored as binary matrix ‘0–1′, where ‘1′ indicated presence of a marker, while ‘0′ indicated an absence. The *Acc65*I/*Mse*I (A) platform provides information about DNA sequence and methylation changes collectively, and the *Kpn*I/*Mse*I (K) platform is responsible for delivering information only about DNA sequence changes; thus, juxtaposing the both platforms allows us to extract only methylation markers M (*Acc65*I/*Mse*I-*Kpn*I/*Mse*I). Instead of *Mse*I, selective primers that targeted the sequences of mobile elements were used in performing PCR reactions in the presence of CpG, CpXpG, and CpXpX (methylation) primers. This allowed us to reflect on changes that TEs might have caused concerning DNA methylation. Based on the properties of the restriction enzymes (*Acc65*I and *KpnI*) that cut DNA sequence 5′-GGATCC-3′, it was possible to estimate changes in specific methylation contexts, symmetric—CG and CHG—and asymmetric—CHH—using special combinations of selective primers (Supplementary Table S1) (Orłowska and Bednarek 2020). Profiles obtained via the MSTD approach were used to assess qualitative and quantitative molecular characteristics: tissue culture induced variation (TCIV) and sequence variation (SV), demethylation (DMV), and de novo methylation (DNMV). In the metAFLP approach, the donor plant DNA profiles were used as reference for the respective regenerants’ profiles generated in two AFLP platforms. The comparison of the profiles in two platforms was used for the evaluation of quantitative characteristics of variation. The results of the metAFLP quantitative characteristics have been included in the Excel spreadsheet (File S2).

### 4.5. Statistical Analysis

A The percentage of polymorphic loci (*%P*) generated by MSTD for donor plants and regenerants and the marker system informativeness evaluated by Shannon’s information index (*I*) was assessed used GenAlEx6.501 (Excel add-in software) [130].

A one-way analysis of variance (ANOVA) was applied to the RP-HPLC results. Two-way ANOVA was conducted for the molecular characteristics for MSTD. The presence of outliers was evaluated via visual inspection of box-plots, Cook’s distances, and Leverage coefficients. The Shapiro–Wilk tests were performed to test for the normality. Homogeneity assumption of ANOVA was verified using the Levene’s test of equality of error variance. Interaction and simple main effects were tested. The SPSS v 25 software [131] was used for ANOVA.

Regression analysis for three models A (CHH_SV: CHH_DNM, CHH_DNMV, CHH_DMV * CHH_DNMV), B (CHG_SV: CHG_DNM, CHG_DNMV, CHG_DMV * CHG_DNMV), and C (CG_SV: CG_DNM, CG_DNMV, CG_DMV*CG_DNMV), including regression assumption testing, as well as linear regression analysis (SV: Global DNA methylation based on RP-HPLC data), was conducted in the SPSS v 25 software.

Automated linear regression analysis combining models A, B, C, and D were conducted in the SPSS software using default settings.

## Figures and Tables

**Table 1:** Percentage of polymorphic loci (*%P*) and Shannon’s information index (*I*) for MSTD data amplified from plant materials.

MSTD Platform	*Kpn*I/*Mse*I (K)		*Acc65*I/*Mse*I-*Kpn*I/*Mse*I (M)	
Plant Material ^1^	*%P*	*I*	*%P*	*I*
D	30.88	0.167	47.03	0.248
R68	41.93	0.138	56.94	0.230
R69	40.51	0.141	53.54	0.220
R70	41.08	0.134	54.39	0.224
R72	38.81	0.128	52.97	0.223
*Mean*	38.64	0.142	52.97	0.229
*SE*	2.01	0.004	1.63	0.004

^1^ D-donor plants grouped together; R68–R72-regenerants obtained from successive donor plants; *Kpn*I/*Mse*I (K) and *Acc65*I/*Mse*I-*Kpn*I/*Mse*I (M) MSTD platforms; SE—standard error of the mean.

**Table 2:** The MSTD characteristics related to regenerants (R68–R72) obtained from various donor plants and analyzed within different TE families (TE).

	Molecular Characteristics (%)			
Regenerants	TCIV	SV	DMV	DNMV
R68	24.23	13.42	3.40	1.94
R69	20.79	10.42	3.64	1.43
R70	26.90	14.00	4.04	2.40
R72	20.27	11.52	3.41	0.93
	Molecular characteristics (%)			
TE family/mobile element/class	TCIV	SV	DMV	DNMV
CACTA/*Balduin* (II-DNA transposon)	30.49	17.15	4.88	1.96
LARD/*Sukkula/*(I-retrotransposon)	14.65	7.69	2.57	0.95
TRIM/Cassandra/(I-retrotransposon)	33.32	16.78	3.67	1.97
Ty1-*copia/BARE-1/*(I-retrotransposon)	14.75	9.99	1.78	1.06
Ty1-*gypsy*/BAGY-1//(I-retrotransposon)	16.79	7.38	4.83	2.40

**Table 3:** The arrangement of simple main effects describing differences in mean sequence variation scores of the regenerant groups, derived from four donor plants by TE families evaluated based on univariate tests.

TE Family/Mobile Element/Class		Sum of Squares	df	Mean Square	*F* ^1^	Sig.	Partial *η*^2^
CACTA/*Balduin* (II-DNA transposon)	Contrast	62.440	3	20.813	4.121	0.007	0.032
	Error	1919.203	380	5.051			
LARD/*Sukkula*/(I-retrotransposon)	Contrast	70.068	3	23.356	4.624	0.003	0.035
	Error	1919.203	380	5.051			
TRIM/*Cassandra*/(I-retrotransposon)	Contrast	375.049	3	125.016	24.753	0.000	0.163
	Error	1919.203	380	5.051			
Ty1-copia/*BARE-1/(*I-retrotransposon)	Contrast	251.187	3	83.729	16.578	0.000	0.116
	Error	1919.203	380	5.051			
Ty1-*gypsy*/BAGY-1//(I-retrotransposon)	Contrast	157.457	3	52.486	10.392	0.000	0.076
	Error	1919.203	380	5.051			

^1^ Each *F* tests the simple effects of the donor within each level combination of the other effects shown. These tests are based on the linearly independent pairwise comparisons among the estimated marginal means. Computed using α = 0.05.

**Table 4:** The arrangement of simple main effects describing differences in mean sequence variation scores of the regenerant groups, derived from four donor plants by TE families, was evaluated based on univariate tests at α = 0.05.

Regenerant Group		Sum of Squares	df	Mean Square	*F* ^1^	Sig.	Partial *η*^2^
R68	Contrast	1364.547	4	341.137	67.545	0.000	0.416
	Error	1919.203	380	5.051			
R69	Contrast	2231.930	4	557.982	110.480	0.000	0.538
	Error	1919.203	380	5.051			
R70	Contrast	2100.922	4	525.230	103.995	0.000	0.523
	Error	1919.203	380	5.051			
R72	Contrast	1797.460	4	449.365	88.974	0.000	0.484
	Error	1919.203	380	5.051			

^1^ Each *F* tests the simple effects of mobile elements belonged to various TE families within each level combination of the other effects shown. These tests are based on the linearly independent pairwise comparisons among the estimated marginal means.

**Table 5:** Multiple regression results verifying whether DNM and DNMV in different DNA sequence contexts explain sequence variation related to the respective asymmetric and symmetric contexts without focusing on the role of groups of regenerants derived from D68, D69, D70, and D72 donor plants.

Model	B	95% CI for B		SE B	*β*	R^2^*_adj_*
		LL	UL			
CHH_SV(CHH_DMV, CHH_DNMV, CHH_DMV x CHH_DNMV)) ^1^						0.166
constant	3.951 ***	3.5	4.4	0.23		
CHH_DMV	0.671 ***	0.51	0.83	0.082	0.414	
CHH_DNMV	1.164 ***	0.78	1.554	0.918	0.327	
CHH_DMV x CHH_DNMV	–0.394 ***	–0.59	–0.019	0.103	–0.223	
CHH_DMV x CHH_DNMV	–0.394 ***	–0.59	–0.019	0.103	–0.223	
CHG_SV(CHG_DMV_CHG_DNMV, CHG_DMV x CHG_DNMV))						0.162
constant	1.931 ***	1.61	2.26	0.165		
CHG_DMV	0.777 ***	0.53	1.03	0.127	0.323	
CHG_DNMV	1.135 ***	0.72	1.55	0.213	0.291	
CHG_DMV x CHG_DNMV	0.17	–0.33	0.67	0.254	0.037	
CG_SV(CG_DMV, CG_DNMV, CG_DMV x CG_DNMV))						0.198
constant	2.611 ***	2.31	2.91	0.151		
CG_DMV	0.732 ***	0.53	0.93	0.101	0.413	
CG_DNMV	0.903 ***	0.63	1.18	0.139	0.413	
CG_DMV x CG_DNMV	–0.268 ***	–0.42	–0.12	0.075	–0.266	

^1^ due to the Durbin–Watson statistics d = 0.69 the coefficients are not reliable. *** significance at *p* < 0.0005. x - interaction

**Table 6:** Regression results verifying whether DNM and DNMV in different DNA sequence contexts explain sequence variation related to the respective TE families.

Mobile Element/TE Family	Model	*F*(3.76)	*p* ^1^	R^2^*_adj_*	Durbin–Watson Statistics
*Balduin/*CACTA	data	data			
	CHH_SV:CHH_DMV, CHH_DNMV, CHH_DMV x CHH_DNMV	0.021	0.996	0	0.759
	CHG_SV:CHG_DMV, CHG_DNMV, CHG_DMV x CHG_DNMV	0.523	0.668	0.02	1.985
	CG_SV:CG_DMV, CG_DNMV, CG_DMV x CG_DNMV	0.394	0.758	0	1.083
*Sukkula*/LARD	CHH_SV:CHH_DMV, CHH_DNMV, CHH_DMV x CHH_DNMV	1.273	0.29	0.01	-
	CHG_SV:CHG_DMV, CHG_DNMV ***, CHG_DMV x CHG_DNMV	6.371	0.001	0.169	1.289
	CG_SV:CG_DMV, CG_DNMV, CG_DMV*CG_DNMV	6.782	**0.0005**	0.18	1.534
*Cassandra*/TRIM	CHH_SV:CHH_DMV, CHH_DNMV, CHH_DMV x CHH_DNMV	3.157	**0.029**	0.076	0.083
	CHG_SV:CHG_DMV, CHG_DNMV, CHG_DMV x CHG_DNMV	4.04	**0.01**	0.104	1.947
	CG_SV:CG_DMV, CG_DNMV, CG_DMV x CG_DNMV	3.056	**0.033**	0.072	1.644
*BARE-1*/Ty1-*copia*	CHH_SV:CHH_DMV, CHH_DNMV, CHH_DMV x CHH_DNMV	0.893	0.449	0	-
	CHG_SV:CHG_DMV, CHG_DNMV, CHG_DMV x CHG_DNMV	0.324	0.8	0	-
	CG_SV:CG_DMV, CG_DNMV, CG_DMV x CG_DNMV	0.444	0.51	0	-
*BAGY-*1/Ty3-*gypsy*	CHH_SV:CHH_DMV, CHH_DNMV *, CHH_DMV*CHH_DNMV	5.6	**0.002**	0.149	0.466
	CHG_SV:CHG_DMV ***, CHG_DNMV ***, CHG_DMV x CHG_DNMV	38.106	**0.0005**	0.585	2.092
	CG_SV:CG_DMV, CG_DNMV, CG_DMV x CG_DNMV	11.387	**0.0005**	0.283	1.88

^1^*p*—significance of the model; *** or * indicate significance of independent variables at *p* = 0.0005 or 0.05, respectively. x - interaction

## Data Availability

Not applicable.

## Supplementary Materials

The following are available online at https://www.mdpi.com/article/10.3390/ijms22136783/s1, Table S1: Oligonucleotide sequences for MSTD approach. File S1: Data concerning global DNA methylation for regenerants R68-R72 and donor plants (D68-D72). File S2: Full dataset for MSTD characteristics for regenerants derived from D68-D72 donor plants. File S3: Raw images of polyacrylamide gels, from which two sections of Figure 2a,b are presented in the manuscript.

## Abbreviations

BAP	6-benzylaminopurine
IRAP	inter-retrotransposon amplified polymorphism
LARD	large retrotransposon derivatives
LTRs	long terminal repeats
metAFLP	methylation sensitive amplified fragment length polymorphism
MSTD	methyl-sensitive transposon display
REMAP	retrotransposon-microsatellite amplified polymorphism
SSAP	sequence-specific amplified polymorphism
SV	sequence variation
TEs	transposable elements
TEs	transposable elements
TRIM	terminal-repeat retrotransposons in miniature
