# Supplementary material for: Androgenic-Induced Transposable Elements Dependent Sequence Variation in Barley
Source: Int J Mol Sci. 2021 Jun 24;22(13):6783. doi: 10.3390/ijms22136783 (PMC8268840; doi:10.3390/ijms22136783)
Supplement: Supplementary file 1 [file ijms-22-06783-s001.zip › Table_S1.pdf]

Androgenic-induced transposable elements dependent sequence variation in barley

Renata Orłowska[1*], Katarzyna A. Pachota[2], Wioletta Dynkowska[1], Agnieszka Niedziela[1], Piotr T. Bednarek[1]

[1]Department of Plant Physiology and Biochemistry, Plant Breeding and Acclimatization Institute – National Research Institute, Radzików, 05-870 Błonie, Poland

[2]Department of Plant Physiology and Biochemistry, Plant Breeding and Acclimatization Institute – National Research Institute, Radzików, 05-870 Błonie, Poland; Currently does not work at the Institute

Corresponding author: r.orlowska@ihar.edu.pl;

Supplementary Table 1 Oligonucleotide sequences for MSTD approach based on metAFLP.

metAFLP oligomer	
Adaptors	Sequence 5'→3'
Ad1 *Acc65*I	CTC GTA GCA TGC GTA CA
Ad2 *Acc65*I	GTA CTG TAC GCA TGC TAC
Ad1 *KpnI*	CTC GTA GCA TGC GTA CAG TAC
Ad2 *KpnI*	TGT ACG CAT GCT AC
Ad1 *MseI*	TAC TCA GGA CTC ATC
Ad2 *MseI*	GAC GAT GAG TCC TGA G
Preselective primers	
Presel *Acc56*I/*KpnI*	GCA TGC GTA CAG TAC C
Presel *MseI*	GAT GAG TCC TGA GTA AC
Selective primers	
Oligonucleotide targeting sequences containing a methylation motif (metAFLP)	Sekwencja 5'→3'
CpG-GCA	CA TGC GTA CAG TAC CGC A
CpXpG-AGA	CA TGC GTA CAG TAC CAG A
CpXpX-TAA	CA TGC GTA CAG TAC CTA A
Oligonucleotide targeting transposable element sequences (radiolabeled with P^{32}) (MSTD)	5'→3'
BARE-1-5980 (Ty1-*copia*)	CTA GGG CAT AAT TCC AAC AA
BARE-1-C0700 (Ty1-*copia*)	ACA CAC AAA GCA TTC CTC CGC
BAGY-1-C2043 (Ty3-*gypsy*)	TCGTCG CCG GCG TCA TCT CC
BAGY-1-C0651 (Ty3-*gypsy*)	CTA TAC TTG ATT AGG GTT CCA GGG
Sabrina-C0945 (Ty3-*gypsy*)	GCA AGC TTC CGT TTC CGC
Sukkula E0228 (LARD)	GGA ACG TCG GCA TCG GGC TG
Sukkula 91673 (LARD)	TGT GAC AGC CCG ATG CCG ACGTTCC
Balduin (CACTA)	CAG CTA GCA GAC AAC AAG GA
CASP-124R (CACTA)	AAA GGG GAA CAC GAG AAG A
CASP-1F (CACTA)	GAT GAA GAA GAA GAG AAC GA

CASP-1R (CACTA)	CCC AAA TGA AGT GAA AAC C
CAS AY16F (TRIM)	CAT TCG AAC AGA ACT CCC A
CAS AY16R (TRIM)	CAC ATA CAC CAC CAC TCA
CAS 978 (TRIM)	GGT GTG TCC GGG GCG TTA CA
CAS 979 (TRIM)	CCG GGA GCC CAT TCG AAC
